# Indentation of a surface-stiffened elastic substrate

**DOI:** 10.1038/s41598-018-34540-2

**Published:** 2018-11-14

**Authors:** A. P. S. Selvadurai

**Affiliations:** Department of Civil Engineering and Applied Mechanics, McGill University, Montreal, QC H3A 0C3 Canada

## Abstract

The paper deals with the problem of the indentation of a substrate in the form of an isotropic elastic halfspace that is reinforced at the surface by a bonded layer, which has flexibility characteristic of a Poisson-Kirchhoff-Germain thin plate. The presence of the reinforcing layer changes the characteristics of the indentation problem, in that the techniques that are applicable for the solution of the direct indentation of the halfspace cannot be applied to develop a load-displacement relationship for the indenter. The integro-differential equation governing the indentation of the surface reinforced halfspace is solved using a discretization approach and numerical results are presented to demonstrate the influence of the stiffness of the reinforcing layer on the Boussinesq indention problem.

## Introduction

The indentation of elastic materials provides a means of determining their material properties through the interpretation of a load-displacement response. Indenters of different contact shapes are used in such investigations and the customary devices employed in the experiments usually focus on spherical and cylindrical indenters with a flat base. The problem can be quite complicated if the elastic material is fully anisotropic, in which case it is unrealistic to expect that all 21 elastic constants can be determined from a single load-displacement measurement, although manufacturers of well known nano-indentation devices promote them as a panacea for determining elasticity properties of any material at any scale. Some progress can be made when the material being investigated is homogeneous and isotropic. In this case, the well known Hertz^1^ solution for the frictionless indentation of a halfspace by a sphere and the Boussinesq^2^ solution for the frictionless indentation of a halfspace by a flat circular indenter (see also Harding and Sneddon^3^) can be used to good effect in the determination of the elasticity properties. Even in the case of material isotropy, the result of an indentation test provides only an estimate of the combined influences of the elastic constants *G* (the shear modulus) and *ν* (Poisson’s ratio) and additional information such as the surface depression exterior to the indenter needs to be determined if the separate values of the elastic constants are needed. Contact mechanics is an extensively researched topic and the reader is referred to the classical treatises and articles by Galin^4^, Ufliand^5^, Lur’e^6^, Kikuchi and Oden^7^, Goodman^8^, de Pater and Kalker^9^, Duvaut and Lions^10^, Selvadurai^11^, Gladwell^12^, Johnson^13^, Panagiotopoulos^14^, Klarbring *et al*.^15^, Curnier^16^, Raous *et al*.^17^, Persson^18^, Aleynikov^19^, Willner^20^, Selvadurai and Atluri^21^, Paggi *et al*.^22^, Selvadurai and Katebi^23^ and Barber^24^ for significant historical developments and up-to-date compilations of the subject. Many of the classical treatments in contact mechanics focus on contact problems related to isotropic elastic media. In these, the basic approaches used in the formulation of the governing integral equations can be extended to include transversely isotropic elastic media and indenters with elliptical planforms, where the surface deflection profiles, determined during testing through laser scanning and digital image correlation techniques, can be used to establish plausible estimates of transverse isotropy. In connection with the problem examined in this paper, the complete analysis of a contact problem will involve an elastic layer where the thickness of the layer is finite and complete continuity of displacements and tractions are enforced at the interface between the elastic layer and the underlying halfspace. In such an approach, no assumptions are made with regard to the modelling of the elastic layer as a structural element. References to classical studies of layered systems related to the above class of problems by Burmister, Westergaard, and others and extensive references to these studies are given by Ling^25^, Bufler^26^, Selvadurai^11^, Gladwell^12^, Aleynikov^19^ and in a number of articles that deal with layered systems as applied to the mechanics of road transportation structures. The studies by Argatov and Sabina^27,28^ that also address, respectively, (i) a spherical indenter contact problem for a transversely elastic halfspace reinforced with an elastic membrane using an asymptotic modelling approach that is able to recover the case of an inextensible membrane (a generalized problem of the surface indentation of a halfspace reinforced by an embedded inextensible elastic membrane is examined by Selvadurai^29^), and (ii) the problem of the point indentation of an elastic half-space reinforced with a bonded elastic plate and comparisons with the asymptotic model of small-scale indentation of an elastic layer bonded to an elastic half-space. Of related interest is the study by Constantinescu *et al*.^30^, who develop a semi-analytical approach for the problem of axisymmetric indentation of a multi-layered elastic halfspace. This area of research has to its credit several thousand articles by leading elasticians and a comprehensive literature review is beyond the scope of this paper.

Friction between the indenter and the elastic material is rarely discussed in the literature dealing with the interpretation of the indentation response. Analytical studies of frictional contact problems are relatively scarce when processes such as finite friction and interface plasticity are incorporated in the analysis. Although the preceding references also contain citations of frictional contact problems, the articles by Spence^31,32^, Paggi *et al*.^22^ and Selvadurai^33^ can be consulted to determine the various approaches to the formulation and solution of frictional contact problems.

## The Contact Problem

**Figure 1:**
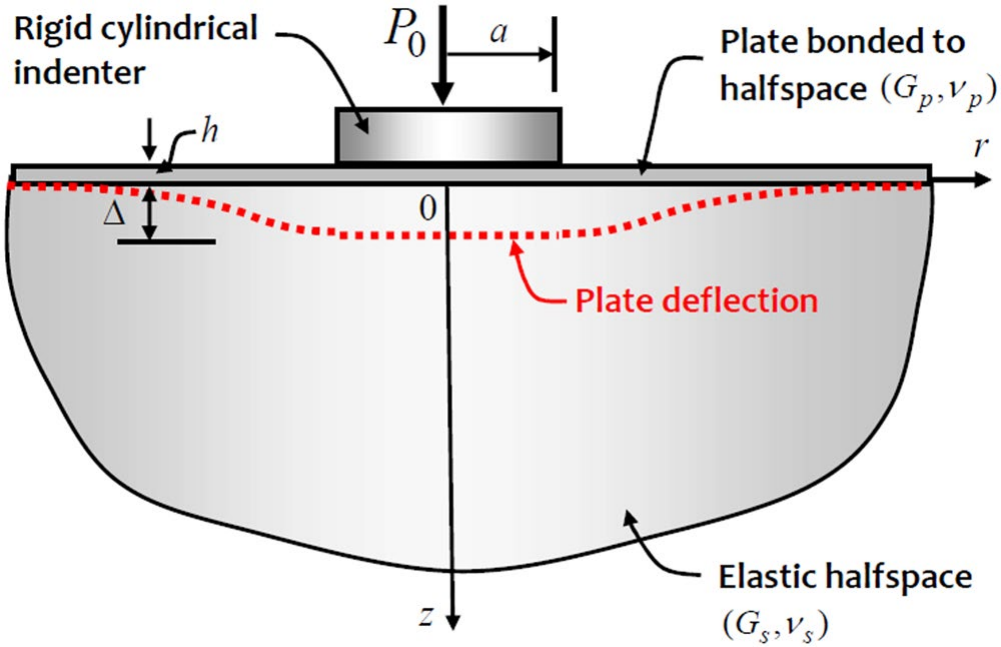
Indentation of a flexible plate bonded to an elastic halfspace.

The theme of this paper is to investigate indentational contact problems that will be relevant to the study of surface stiffened substrates. The specific problem modelled in the paper relates to a halfspace region where the surface is reinforced with a thin elastic layer, the flexibility characteristics of which can be modelled by the Germain-Poisson-Kirchhoff thin plate theory. The extensions of the work to include other plate models, such as the Reissner or Mindlin plate theories and constrained elastic layers such as the one proposed by Vlazov and Leontiev^34^ are possible, but these will be investigated in future work. The problem examined relates to the Boussinesq indentation of a thin plate that is bonded to an isotropic elastic halfspace region (Fig. 1).

The deflected shape of a plate that is bonded to a halfspace region can be obtained using standard techniques (Selvadurai^11^) and, due to the axisymmetric nature of the problem, the differential equation governing the axisymmetric state of bending of the plate is given by (Selvadurai^11^, Gladwell^12^, Selvadurai and Dumont^35^).

1 $$D{\tilde{\nabla }}^{2}{\tilde{\nabla }}^{2}w(r)+\frac{2{G}_{s}}{\pi (1-{\nu }_{s})r}\frac{d}{dr}{\int }_{0}^{r}\frac{s}{\sqrt{{s}^{2}-{r}^{2}}}(\frac{d}{ds}{\int }_{0}^{s}\frac{r\,w(r)\,dr}{\sqrt{{s}^{2}-{r}^{2}}})ds=p(r)$$

where *w*(*r*) is the plate deflection, $D(\,=\,{G}_{P}{h}^{3}/6(1-{\nu }_{P}^{2}))$ is the flexural rigidity of the plate, *G*_*i*_, *ν*_*i*_ (*i* = *s*, *p*) are the elastic constants of the halfspace and plate materials, *h* is the plate thickness, *p*(*r*) is the externally applied load and

2 $${\tilde{\nabla }}^{2}=\frac{{d}^{2}}{d{r}^{2}}+\frac{1}{r}\frac{d}{dr}$$

is the one-dimensional radially symmetric form of Laplace’s operator. The indentation of the surface stiffened elastic halfspace by a Boussinesq indenter will impose the following mixed boundary conditions on the plate:

3 $$w(r)={\rm{\Delta }}\,;\,0\le r\le a$$

4 $$p(r)=0\,;\,a\le r < \infty $$

In addition, regularity conditions can be prescribed on the displacement w(r) and its first derivative, as r → ∞ to ensure well-posedness of the contact problem.

## The Discretization Approach

**Figure 2:**
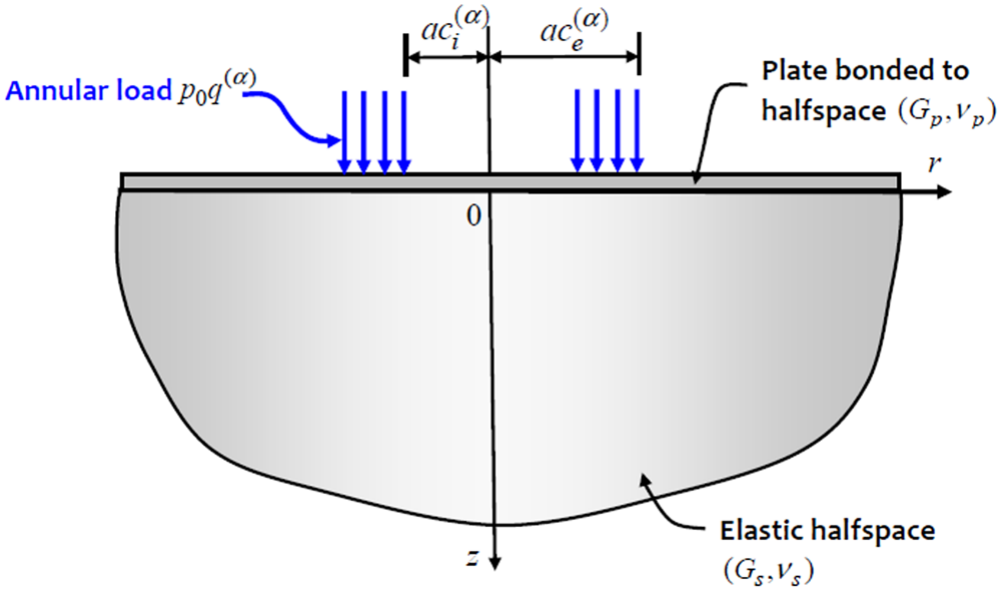
Annular loading of a plate bonded to an elastic halfspace.

Due to the integro-differential nature of the governing equation, it is unlikely that an explicit solution can be obtained for the mixed boundary value problem posed above. The discretization approach adopted in this paper is to consider that the contact stress between the indenter and the flexible plate is composed of a finite number of annular regions of constant intensity (Fig. 2).

In order to use a non-dimensional formulation, we assume that the annular region of the uniform contact stress *q*^(*α*)^ between the rigid indenter and the elastic plate is given by ${q}^{(\alpha )}={p}_{0}{\bar{Q}}^{(\alpha )}$ where ${\bar{Q}}^{(\alpha )}$ is a non-dimensional value of the annular stress and *p*_0_ is the average indentation stress given by

5 $${p}_{0}=\frac{{P}_{0}}{\pi {a}^{2}}$$

where *P*_0_ is the axial force applied to the indenter. The *annular loading problem* can be solved using Hankel transform techniques and the solution can be presented in the form

6 $${\bar{W}}^{A}(\rho )=\frac{w(r)}{{p}_{0}a/{G}_{s}}={F}^{A}({\bar{Q}}^{(\alpha )},{c}_{i}^{(\alpha )},{c}_{e}^{(\alpha )},\rho )$$

where ${c}_{i}^{(\alpha )}$ and ${c}_{e}^{(\alpha )}$ are, respectively, the non-dimensional values of the internal and external radii of the annular loading (Fig. 2) of non-dimensional stress intensity ${\bar{Q}}^{(\alpha )}$, *ρ* = *r*/*a* and the function ${F}^{A}({\bar{Q}}^{(\alpha )},{c}_{i}^{(\alpha )},{c}_{e}^{(\alpha )},\rho )$ is given by

7 $${F}^{A}({\bar{Q}}^{(\alpha )},{c}_{i}^{(\alpha )},{c}_{e}^{(\alpha )},\rho )={\int }_{0}^{\infty }{\bar{Q}}^{(\alpha )}(\frac{3-4{\nu }_{s}}{4-4{\nu }_{s}})(\frac{{c}_{e}^{(\alpha )}{J}_{1}(\xi {c}_{e}^{(\alpha )})-{c}_{i}^{(\alpha )}{J}_{1}(\xi {c}_{i}^{(\alpha )})}{\xi [1+{\xi }^{3}{\rm{\Omega }}]}){J}_{0}(\xi \rho )d\xi $$

where

8 $${\rm{\Omega }}=\frac{(3-4{\nu }_{s})}{24(1-{\nu }_{s})(1-{\nu }_{p})}(\frac{{G}_{p}}{{G}_{s}}){(\frac{h}{a})}^{3}$$

The parameter Ω is indicative of the relative stiffness of the plate-elastic halfspace system. For example, (i) when Ω → 0 (either as a result of *h* → 0 or *G*_*p*_ → 0), (6) reduces to the solution for the problem of the loading of a halfspace region by an annular load, and (ii) when Ω → ∞ (notably as a result of *G*_*p*_ → ∞, since for a finite *G*_*p*_, a rigid plate model cannot be recovered by setting *h* → ∞, see e.g. Selvadurai^11^), the integral (7) reduces to zero and the displacement of a plate of infinite relative stiffness bonded to a halfspace region and subjected to a finite load gives rise to a null displacement field.

For the solution of the axisymmetric contact problem using a discretization approach it is also necessary to consider the situation where the central value of the contact stress is modelled by the case where the plate is subjected to a circular load of uniform intensity *p*_0_ *q*^(0)^. This solution can be recovered from (6) and (7) but, for convenience, we shall present the result as

9 $${\bar{W}}^{(0)}(\rho )=\frac{w(r)}{{p}_{0}a/{G}_{s}}={F}^{C}({\bar{Q}}^{(0)},{c}^{(0)},\rho )$$

where

10 $${F}^{C}({\bar{Q}}^{(0)},{c}^{(0)},\rho )={\int }_{0}^{\infty }{\bar{Q}}^{(0)}(\frac{3-4{\nu }_{s}}{4-4{\nu }_{s}})(\frac{{c}^{(0)}{J}_{1}(\xi {c}^{(0)})}{\xi [1+{\xi }^{3}{\rm{\Omega }}]}){J}_{0}(\xi \rho )d\xi $$

We assume that the contact stress distribution beneath the rigid indenter can be represented as *n* annular regions of equal area.

As indicated by Selvadurai^11^ and recently applied by Selvadurai and Katebi^23^ to contact problems involving incompressible inhomogeneous elastic media, the preceding developments are used to evaluate the deflections of the plate subjected to a central uniform load ${\bar{Q}}^{(0)}$ and the annular loads ${\bar{Q}}^{(\alpha )}$. In particular, the deflections are evaluated at the central uniform load ${\bar{Q}}^{(0)}$ and at a central position within the annular loading region ${\bar{Q}}^{(\alpha )}$. If we denote the non-dimensional deflections of the plate due to these loadings at the specified positions by ${\bar{W}}^{(0)}\,,{\bar{W}}^{(1)}\,,{\bar{W}}^{(2)},\,{\bar{W}}^{(3)},\mathrm{.....},{\bar{W}}^{(n)}$, the corresponding set of equations for the plate deflections can be written as

11 $$\begin{array}{rcl}{\bar{W}}^{(0)} & = & {C}_{00}{\bar{Q}}^{(0)}+{C}_{01}{\bar{Q}}^{(1)}+{C}_{02}{\bar{Q}}^{(2)}+{C}_{03}{\bar{Q}}^{(3)}+\ldots \ldots \ldots \ldots \ldots \ldots \ldots \ldots +{C}_{0n}{\bar{Q}}^{(n)}\\ {\bar{W}}^{(1)} & = & {C}_{10}{\bar{Q}}^{(0)}+{C}_{11}{\bar{Q}}^{(1)}+{C}_{12}{\bar{Q}}^{(2)}+{C}_{13}{\bar{Q}}^{(3)}+\ldots \ldots \ldots \ldots \ldots \ldots \ldots \ldots +{C}_{1n}{\bar{Q}}^{(n)}\\ {\bar{W}}^{(2)} & = & {C}_{20}{\bar{Q}}^{(0)}+{C}_{21}{\bar{Q}}^{(1)}+{C}_{22}{\bar{Q}}^{(2)}+{C}_{23}{\bar{Q}}^{(3)}+\ldots \ldots \ldots \ldots \ldots \ldots \ldots \ldots +{C}_{2n}{\bar{Q}}^{(n)}\\ {\bar{W}}^{(3)} & = & {C}_{30}{\bar{Q}}^{(0)}+{C}_{31}{\bar{Q}}^{(1)}+{C}_{32}{\bar{Q}}^{(2)}+{C}_{33}{\bar{Q}}^{(3)}+\ldots \ldots \ldots \ldots \ldots \ldots \ldots \ldots +{C}_{3n}{\bar{Q}}^{(n)}\\ \ldots \ldots \ldots  & = & \ldots \ldots \ldots \ldots \ldots \ldots \ldots \ldots \ldots \ldots .\\ \ldots \ldots \ldots  & = & \ldots \ldots \ldots \ldots \ldots \ldots \ldots \ldots \ldots \ldots .\\ {\bar{W}}^{(n)} & = & {C}_{n0}{\bar{Q}}^{(0)}+{C}_{n1}{\bar{Q}}^{(1)}+{C}_{n2}{\bar{Q}}^{(2)}+{C}_{n3}{\bar{Q}}^{(3)}+\ldots \ldots \ldots \ldots \ldots \ldots \ldots \ldots +{C}_{nn}{\bar{Q}}^{(n)}\end{array}$$

This result can be written in the non-dimensional form

12 $$\{{\bar{{\bf{W}}}}^{(\alpha )}\}=[{\bf{C}}]\{{\bar{{\bf{Q}}}}^{(\alpha )}\}\,;\,(\alpha =0,1,2,3,\mathrm{.......},n)$$

and the conversion to the physical variables is accomplished through the relationships

13 $${W}^{(\alpha )}=\frac{{p}_{0}a}{{G}_{s}}{\bar{W}}^{(\alpha )}\,;\,{q}^{(\alpha )}=\frac{{P}_{0}}{\pi {a}^{2}}{\bar{Q}}^{(\alpha )}$$

In the case of the indentation of the flexible plate by a rigid circular indenter

14 $${\bar{W}}^{(\alpha )}=\frac{{\rm{\Delta }}}{a}={\rm{const}}{\rm{.}}$$

and the additional equilibrium equation required for closure is

15 $$\sum _{\alpha =0}^{n}{q}^{(\alpha )}{A}_{(\alpha )}={P}_{0}$$

where ${A}_{(\alpha )}$ corresponds to either the area of the central circular contact region $(\alpha =0)$ or the annular contact regions $\alpha =1,2,3,\mathrm{....},n$. The unknowns (Δ/α) and *q*^(*α*)^ can be determined from (12) and (15).

## Limiting Cases and Numerical Results

In order to evaluate integrals of the type indicated by (7), two quadrature techniques have been used. The first relates to a straightforward Gaussian quadrature technique and the second involves an adaptive quadrature technique using the “*quadgk*” algorithm available in Matlab™. In order to evaluate the accuracy of the results, the results for the case (Ω = 0 and ν_s_ = 0.499) has been compared with the results for the contact problem on an incompressible elastic half-space^23^.

As indicated previously, the limit Ω → 0, corresponds to the case where the plate is absent, but the surface of the halfspace contains an inextensibility constraint imposed by satisfying the kinematic compatibility between the deflections of the plate and the surface of the halfspace. In this case the indentation problem corresponds to a modified form of the Boussinesq indentation by a rigid circular indenter with a flat base but with the inextensibility kinematic constraint, which corresponds to the mixed boundary conditions:

16 $${u}_{z}(r,0)={{\rm{\Delta }}}_{0}\,;\,0\le r\le a$$

17 $${u}_{r}(r,0)=0\,;\,0\le r < \infty $$

18 $${\sigma }_{zz}(r,0)=0\,;\,a < r < \infty $$

The solution of this mixed boundary value problem has been discussed by Selvadurai^36^ and the analysis of the problem entails the reduction of the associated dual integral equations to a single Abel integral equation, which can be solved in exact closed form. Omitting details, it can be shown that when Ω → 0 and in the presence of the inextensibility constraint (17), we obtain

19 $${{\rm{\Delta }}}_{0}=\frac{{P}_{0}(3-4{\nu }_{s})}{16{G}_{s}a(1-{\nu }_{s})}$$

Also, in the limit of an incompressible elastic material, (19) reduces to the classical result

20 $${[{{\rm{\Delta }}}_{0}]}_{v=1/2}=\frac{{P}_{0}}{8{G}_{s}a}$$

By virtue of the analogy between the problem of Kelvin^37^ for the concentrated force located at the interior of an infinite space region and the solution by Boussinesq^2^ for a concentrated normal force acting on the surface of a halfspace (see also, Westergaard^38^, Selvadurai^39^), and in the limit of material incompressibility of the halfspace region, the inextensibility constraint (17) has no influence on the indentational stiffness of the rigid circular indenter. The contact stresses at the base of the rigid circular indenter are uninfluenced by the inextensibility constraint resulting from the limit Ω → 0, and this is given by

21 $${\sigma }_{zz}(r,0)=\frac{{P}_{0}}{2\pi a\sqrt{{a}^{2}-{r}^{2}}}\,;\,0\le r < a$$

As Ω → ∞, the flexible plate becomes infinitely rigid and the analysis yields a trivial result Δ → 0 and *σ*_*zz*_(*r*, 0) → 0; *r* ∈ (0, ∞).

**Figure 3:**
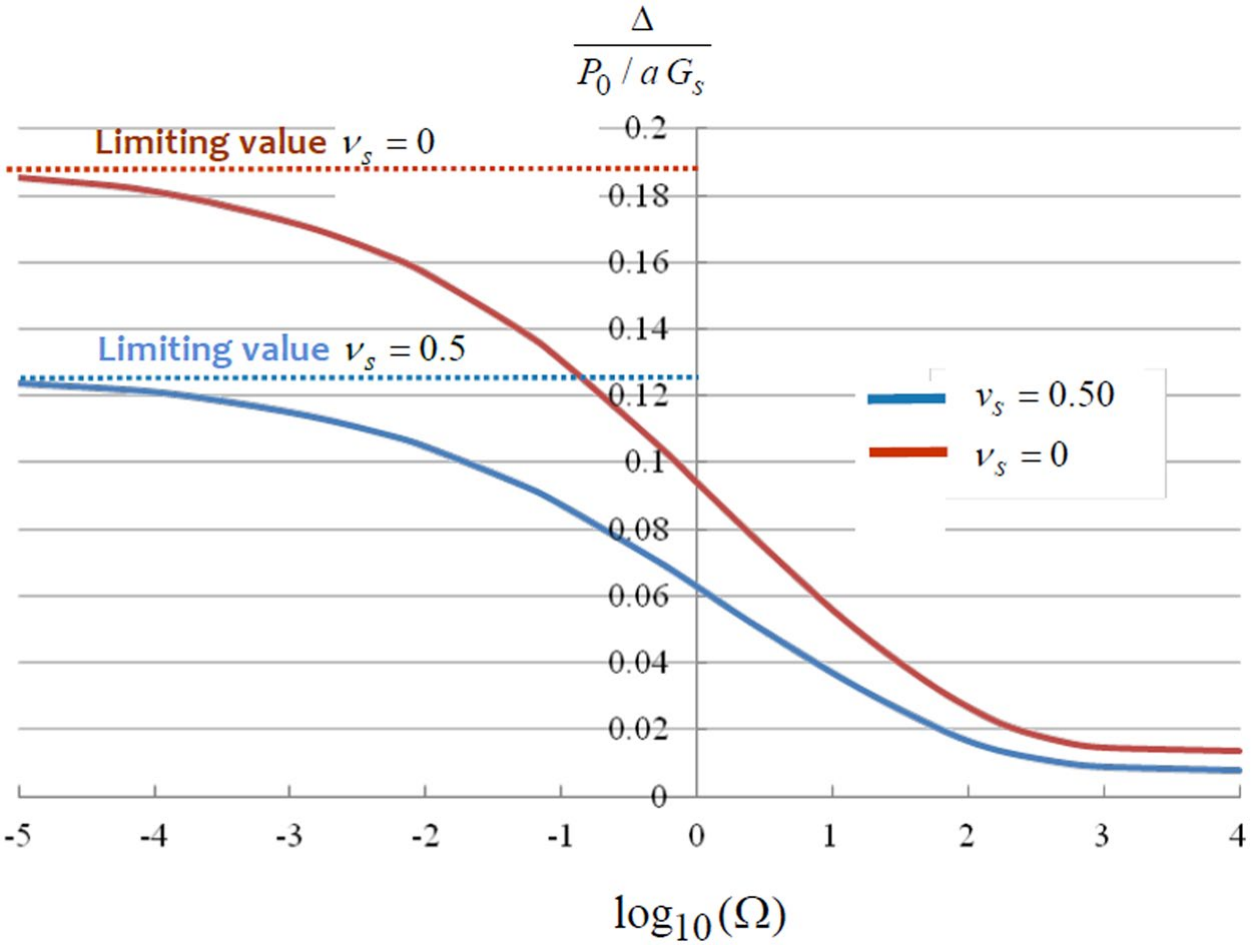
Influence of the relative stiffness of the bonded plate on the indentational displacement.

The analytical discretization procedure outlined here can be used to develop solutions for the stiffness of the rigid circular indenter for arbitrary values of the relative stiffness parameter Ω. Figure 3 illustrates the non-dimensional indentation displacement as a function of the relative stiffness parameter Ω. The results also indicate the influence of the Poisson’s ratio on the rigid displacement of the indenter. When the flexural rigidity of the bonded layer reduces to zero, Ω → 0 and the exact solution is given by (19). The results obtained by the discretization approach give values identical to the exact result; i.e. [Δ_0_/(*P*_0_/*G*_*s*_*a*)]_*ν*=0_ = 0.1875 and [Δ_0_/(*P*_0_/*G*_*s*_*a*)]_*ν*=0.5_ = 0.1250. Due to the approximate computational nature of the discretization procedure, however, the limiting result of the non-dimensional displacement gives a non-zero finite value (less than 10%) as Ω → 10^4^. This residual value can be minimized by increasing the number of discretizations associated with the proposed analysis.

**Figure 4:**
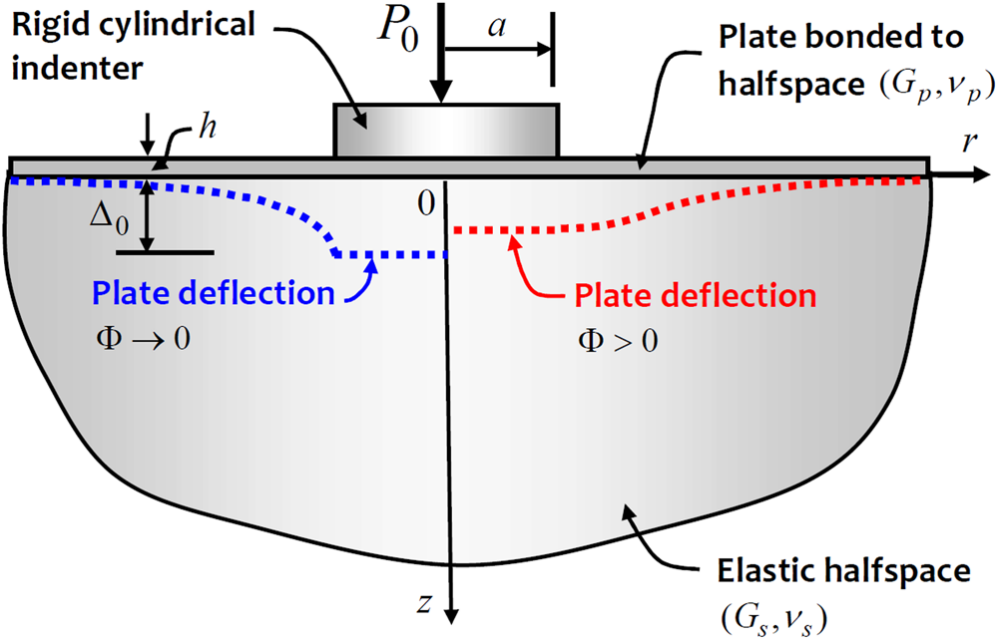
Influence of the relative stiffness of the bonded flexible plate on the surface deflection pattern.

**Figure 5:**
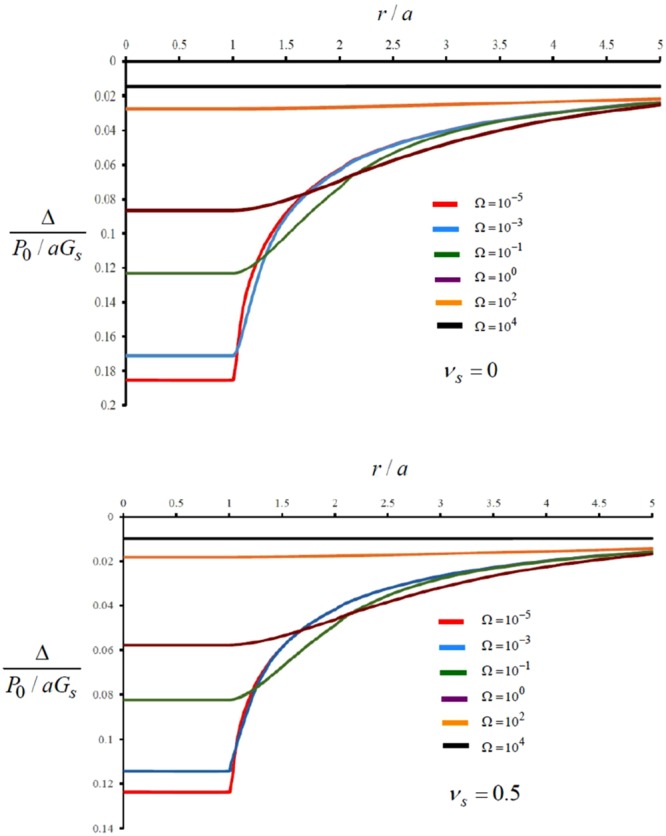
Influence of the relative stiffness of the bonded flexible plate on the surface deformations of the halfspace region.

The discretization technique can also be used to determine the flexural deflections of the surface bonded plate during Boussinesq indentation by a rigid circular indenter. Figure 4 illustrates the typical limiting cases of the surface displacements associated with the indentation. The results obtained from the discretization approach are shown in Fig. 5. The discretization scheme shows results consistent with the limiting cases. Again, as the relative rigidity of the surface reinforcing plate approaches infinity, the rigid displacement approaches a finite value.

## Discussion

The surface reinforcement of substrates is important to several areas of material science and engineering in order to enhance abrasion resistance, minimize deformation at contacts and prevent failure development at contact zones^40^. The assessment of the integrity of the bond between the reinforcing layer and the substrate can be performed using both static and dynamic indentation tests and wave propagation tests. The static indentation test is perhaps the most straight forward and the indentation response can be used to assess the integrity of the bond. This paper presents a study of the indentation response of a surface reinforced solid where there is complete adhesion between the reinforcing layer and the substrate. The representation of the reinforcing layer as a flexible plate is an approximation and can be improved by adopting other models of reinforcing layers that can accommodate both deformations due to flexure and shear deformations. The problem of the contact between an infinite plate on an elastic halfspace is indeed classical and as the author has pointed out, there are classical treatises that investigate all types of advances in this area. The problem of a *finite plate* on an elastic halfspace has been examined using analytical methods, approximate series expansion techniques to accommodate singular stress fields at the boundary of the indenting plate. Variational methods have also been successfully applied to examine the contact problem^41–43^. The indentation problem examined in the paper is certainly out of the ordinary, in that the basic governing equations are of the integro-differential type and the mixed boundary conditions associated with the indentation are applied to this integro-differential equation. The paper demonstrates the application of a discretization method that is amenable to the development of analytical solutions that can be used as benchmarks for the validation of computational approaches based on finite element and boundary element schemes^44,45^. The discretization approach gives accurate results provided the relative rigidity of the surface stiffening layer is finite. This is in keeping with the objectives of the surface stiffening process, which is to provide compliance for the indenting action. A nearly rigid surface stiffening layer can lead to the development of brittle fracture during indentation. If the results of interest focus on estimating stiffness of the indenter, the discretization approach presented in the paper is a satisfactory and reliable technique, even in situations where the shape of the indenter can induce singular stress fields. The procedure can be extended to examine other types of axisymmetric indenters including spherical and paraboloidal shapes.
